# The HIV drug optimization agenda: promoting standards for earlier investigation and approvals of antiretroviral drugs for use in adolescents living with HIV

**DOI:** 10.1002/jia2.25576

**Published:** 2020-08-31

**Authors:** Pablo Rojo, Deborah Carpenter, François Venter, Anna Turkova, Martina Penazzato

**Affiliations:** ^1^ Pediatric Infectious Diseases Unit Department of Pediatrics Hospital 12 de Octubre Universidad Complutense Madrid Spain; ^2^ Maternal and Child Health Branch Division of Global HIV and Tuberculosis Center for Global Health US Centers for Disease Control and Prevention Atlanta GA USA; ^3^ Wits Reproductive Health and HIV Institute University of the Witwatersrand Johannesburg South Africa; ^4^ MRC Clinical Trials Unit at UCL Institute of Clinical Trials & Methodology London United Kingdom; ^5^ HIV Department World Health Organization Geneva Switzerland

**Keywords:** HIV, adolescents, drug optimization, ARV

## Abstract

**Introduction:**

Most clinical trials for new antiretroviral (ARV) agents are conducted among narrowly defined adult populations. Only after safety and efficacy have been clearly demonstrated among adults living with HIV are trials including adolescents, children and infants conducted. This approach contributes to significant delays in the availability of optimal new ARV regimens for infants, children and adolescents. This commentary discusses issues related to the inclusion of adolescents aged 12 to 18 years in initial HIV clinical phase 3 trials of novel antiretrovirals (ARVs) or conducting parallel phase 3 clinical trials among adolescents.

**Discussion:**

The absorption, metabolic and excretion or elimination pathways for drugs do not significantly differ between adolescents and adults. In fact, dosing recommendations for ARVs are the same for adults and adolescents who meet the age and weight criteria. Although conducting clinical trials among adolescents present special challenges (e.g. consenting minors and concerns about trial completion and contraception), these challenges can be addressed to obtain high‐quality trial results. Importantly, new agents and optimized combinations have more favourable dosing schedules and side‐effect profiles and are more effective ARV agents with higher HIV drug resistance thresholds, which would be extremely beneficial to improve outcomes among HIV‐positive adolescents.

**Conclusions:**

Adolescents may not present with significantly different pharmacokinetic characteristics from those in adults. Including HIV‐positive adolescents in phase 3 ARV clinical trials, either with adults or in specific adolescent studies conducted in parallel, would allow adolescents to access promising, more effective treatment for HIV years earlier than with the current stepwise approach.

## 1 INTRODUCTION

Most clinical trials for new antiretroviral drugs (ARVs) are conducted among narrowly defined adult populations. Only after safety and efficacy have been clearly shown in adults do trials move to include HIV‐positive children and adolescents, significantly delaying the availability of ARVs and/or optimized combinations for use among these groups (see Table 1). This stepwise approach also is applied to pregnant or breast‐feeding women and to women of reproductive age who are not receiving contraception. Historical rationale for this approach mostly includes; focus on the differences between adults and children/adolescents as they relate to drugs, developmental variability, complexities of parental involvement and adaptations required in research procedures [35].

**Table 1:** Examples of antiretroviral approvals by the United States Food and Drug Administration and dosing recommendations in adults and adolescents^a^

Drug	Year approved, adults	Dose, adults	Year approved, adolescents	Dose, adolescents
Tenofovir disoproxil fumarate (Viread)	2001	300 mg OD	2010	300 mg OD for ≥12 years and ≥35 kg
Darunavir (Prezista)	2006	600 mg BID	2008	600 mg BID, for ≥40 kg
Bictegravir, only available in a fixed‐dose combination tablet (Biktarvy)Bictegravir 50 mg/emtricitabine 200 mg/tenofovir alafenamide 25 mg	2018	50 mg OD	2019	50 mg OD, for ≥25 kg

^a^ United States Guidelines for the Use of Antiretroviral Agents in Adults and Adolescents with HIV. Available at http://aidsinfo.nih.gov/contentfiles/lvguidelines/AdultandAdolescentGL.pdf.

OD, once a day; BID, twice a day.

The Paediatric Antiretroviral Working Group led by the World Health Organization (WHO), has proposed optimizing research approaches for children and adolescents to speed‐up the availability of ARVs and formulations for these groups [1]. Recently, the United States Food and Drug Administration (US FDA) recommended that ARV drug trials include adolescents (aged 12 to <18 years) in phase 3 clinical trials along with adults, or that a separate phase 3 clinical trial among adolescents be conducted in parallel [2]. Adult trials for tuberculosis [3] and cancer treatment [4] as well as ADVANCE, a recent clinical trial that enrolled HIV‐positive participants aged ≥12 years, have shown the feasibility of including adolescents in adult clinical trials [5].

Adolescence is a period of rapid growth and change. Physical, mental and emotional maturation changes occur as a person transitions into adulthood and begins to take their own care and decision making [6]. Adolescents are a heterogenous population that includes individuals in various stages of emotional, physical, intellectual and sexual development. Gender differences, and environmental factors such as geographical location, socioeconomic and sociodemographic differences, home environment, cultural and social support, play a mayor role in influencing adolescents’ behaviour. Adolescence has been widely associated with risk‐taking behaviour [7]; however, it is also a period for reflection, for generating great ideals and building one’s personality. In this commentary, we defined adolescents as individuals age 12 to 18 years, thus aligning with the US FDA clinical trials recommendation.

UNAIDS 2018 estimates that 1.6 million adolescents aged 10 to 19 years are living with HIV globally. Adolescents also remain vulnerable to acquiring new HIV infection and to have sub‐optimal clinical outcomes [8]. There is a paucity of HIV treatment coverage data for adolescents living with HIV (ALHIV) and where there is data, access and uptake of antiretroviral treatment (ART) is often reported to be lower than among HIV‐positive adults [9]. Better‐tolerated ARVs and ART regimens are needed for ALHIV, to reduce acute and late‐onset side effects, to improve adherence and to ensure long‐term viral suppression. Expanding treatment options for adolescents is a priority, requiring clinical trial generated evidence on new ARVs formulations, dosing options and combination regimens to optimize treatment for this vulnerable population. Not including adolescents in new drug development trials, simplification trials or HIV treatment failure studies will continue to widen the existing ART knowledge gap and further disadvantage this population. This commentary discusses benefits and challenges of including adolescents in clinical phase 3 trials of ARVs or the performance of parallel adolescents’ phase 3 clinical trials irrespective of the mode of HIV acquisition, that is perinatally and sexually acquired.

## 2 DISCUSSION

This section provides the rationale and justification for including adolescents in initial adult clinical phase 3 trials of novel ARVs and combinations or conducting parallel phase 3 clinical trials among adolescents.

### 2.1 No major pharmacokinetic differences

Absorption, hepatic metabolism and renal elimination of drugs in infants and young children are significantly different than in adults, but there are no major pharmacokinetic differences between adolescents and adults [10, 11]. However, bone mineralization, which is associated with bone metabolism and growth may be a physiological difference between adolescents and adults. Bone, which is a metabolic tissue, undergoes rapid changes during adolescence and approximately 80% of adolescents achieve peak bone mass by age 18 years [12]. Tenofovir disoproxil fumarate (TDF) is the ARV most clearly related to decreased bone mineral density in HIV‐positive adults. Data on children and youth are conflicting. Results of a 10‐year follow‐up study in Italy indicate that a TDF‐containing regimen does not decrease the bone mineral density of HIV‐positive youths [13]. However, a longitudinal study in Brazil showed that TDF contributes to decreased bone mineral density among adolescents [14]. Both the US Department of Health and Human Services (DHHS) guidelines and the WHO guidelines recommend the use of TDF at the adult dose in adolescents weighing >35 kg. WHO guidelines also recommend Zidovudine and Abacavir as alternatives to TDF under special circumstances [15, 16, 17].

### 2.2 Similar dosing recommendations for adolescents and adults

Dosing recommendations for ARVs have consistently been the same for adults and adolescents. The DHHS guidelines recommend “adult tablets” for both adults and post‐pubertal adolescents [11, 15]. The DHHS guidelines also recommend using most adult tablets for adolescents with a few exceptions based on weight: for example all adult ARV tablets are allowed for adolescents weighing ≥40 kg, some adult ARV tablets are allowed for adolescents weighing >35 kg, and in some cases, >25 kg [15]. The annex to the updated WHO recommendations on ARV regimens suggests the same dosing for adults and adolescents [16].

Furthermore, the ODYSSEY trial evaluated the use of the adult dolutegravir (DTG) tablet (50 mg) not only in adolescents, but also in children weighing 20 to <40 kg. The results showed that the adult tablets achieved appropriate pharmacokinetic profiles with no safety signal, allowing practical dosing and rapid access to DTG [19].

The DHHS guidelines recommend taking the patient’s sexual maturation rating (SMR) into consideration to select the dosing for ARVs, but neither the WHO 2016 ARV guidelines [17] or the Penta 2019 guidelines [18] even mention SMR. SMR is only mentioned three times in the DHHS Paediatric Guidelines Dosing Annex: dosing for efavirenz (400 mg) has not been evaluated in patients with an SMR 3 or less; for cobicistat/elvitegravir/emtricitabine/tenofovir (brand name, Stribild) which is recommended for adolescents who weigh >35 kg and have SMR 4 to 5; and TDF is recommended for adolescents who weight >35 kg and have SMR 1 to 2. However, the guidelines do not provide different dosing recommendations based on SMR. Practically, healthcare providers consider age and/or weight (vs. SMR) to select the dosing and to initiate ARVs per WHO or DHHS recommendations which have already factored in SMR [15, 17, 34].

### 2.3 Supply chain benefits

Using the same ARV formulations and dosing for adolescents and adults can synergize programme‐level supply chain management. For example new drugs approved for both adolescents and adults can use an existing supply chain system, thereby minimizing inefficiencies caused by setting up a new supply chain system for adolescents only. Using the same dosing, formulations and combinations enhance the effectiveness and efficiency of the supply chain by minimizing the different types of ARVs that a supply chain specialist, a healthcare provider and clients have to manage. Having different types of ARVs for adults and adolescents can overwhelm the healthcare system and providers leading to an overloaded/inefficient supply chain system.

Moreover, using same dosing and formulations for both adolescents and adults streamlines ARVs treatment across populations, facilitating the implementation of treatment guidelines.

### 2.4 Possible challenges to participation of adolescents in clinical trials

Challenges to allowing adolescents to participate in adult clinical trials include recruiting adolescents, lack of treatment adherence and sub‐optimal retention [3, 21]. Obtaining informed consent from adolescents also can be difficult because adolescents typically are required to obtain the approval of an adult caregiver. Adolescents also need specific clinical trial materials that explain the trial using age‐appropriate language. Some clinical trials require female participants to use contraception which may be challenging to assess and to address possibly due to fear of disclosure [36].

### 2.5 Recruiting adolescents into clinical trials

Other types of clinical trials have had challenges recruiting adolescents. For example the participation rate of 15 to 19 year olds in a national clinical trial of cancer drugs in the United States (1997 to 2003) was approximately half of the corresponding rate in children aged <15 years [20]. The study suggested that one of the reasons for low enrolment numbers could be that adolescents exist in a “no‐man’s land” between the worlds of paediatric and adult medical oncology [21]. Enhanced collaboration between paediatric and adult specialists involved in caring for adolescents may improve participation in clinical trials.

Adolescent participation in HIV clinical trials is variable. The recent ADVANCE trial enrolled participants aged ≥12 years in South Africa; after 2 years 1053 patients had undergone randomization, but only 14 patients were <19 years, despite extensive efforts to recruit adolescents [5]. However, in the ODYSSEY trial (PENTA 20), a randomized trial of DTG‐based ART versus the standard of care for children aged <18 years starting first‐line ART or switching to second‐line ART, the targeted number of adolescents was achieved quickly due to higher than expected numbers of adolescent willing to participate. To increase the numbers of younger children in this trial, the researchers had to cap the recruitment of ART‐naïve children who weighed ≥35 kg. Of 708 enrolled participants, 52% were aged >12 years at trial initiation [22].

Collaboration among paediatric, adolescent and adult HIV clinics is crucial. Protocols that not only allow adolescents to be recruited but also identify specific adolescent HIV clinics, could facilitate recruiting adolescents. FDA guidance advises doing these studies in parallel or in the same protocol for adults [2].

### 2.6 Adherence to treatment and follow‐up retention in clinical trials

There are concerns that adolescents have difficulty adhering to medication regimens, which might hinder adherence to study drugs and attendance at follow‐up visits after enrolment [23]. The advanced FDA Paediatric HIV guidance for Industry suggests that adolescents have lower adherence rates than adults [2]. Nevertheless, similar treatment adherence estimates have been reported both overall and by region in a meta‐analysis of adult adherence [24] and in a meta‐analysis of adolescent adherence [25]. Researchers could consider providing additional adherence support to young adolescents participating in trials with adults since young adolescents may have limited ability to self‐care.

Some trials have shown excellent retention of children and adolescents, for example in the BREATHER (PENTA 16) trial, an open‐label, non‐inferiority trial of Efavirenz‐based regimen. The study included 199 participants from 11 countries with a median age of 14 years (interquartile range, 12 to 18 years). Follow‐up in the trial was outstanding, with over 98% of the clinic visits attended up to week 48 [26]. This result demonstrates that excellent follow‐up of ALHIV can be achieved. Researchers are often concerned that including adolescents will skew the results if included in the adult trial. To address these concerns, researchers may analyse the adolescent and the adult population data separately to evaluate the consequences of possible differences. Stratification methods may also be used.

### 2.7 Informed consent

Various ethical and legal complexities may arise when including children and adolescents in clinical trials, especially when obtaining informed consent. Clinical trials must obtain consent from a participant with legal capacity, or from a person with the authority to consent on the participant´s behalf. This means that in most countries, adolescents aged <18 years (except emancipated minors) would need to obtain permission from their caregivers [27] to participate in a study.

Not uncommonly, especially in low‐income countries, adolescents’ caregivers are grandparents or other relatives who are not officially recognized. For example in the ARROW clinical trial in Zimbabwe, a substantial number of potential research participants were orphans (120/400; [30%]), and only 1/120 (0.08%) had a court‐appointed guardian. The Zimbabwean ARROW research team informed the Ethical Committee of this challenges and how this legal requirement could potentially disadvantage this group of children. The Ethical Committee waived the legal guardianship requirement but requested that caregivers sign an informed consent document for the children’s participation [28].

Apart from caregiver consent, adolescents younger than the legal age of consent are required to provide an informed assent to participate in a trial. In addition, if adolescents turn 18 years old during the trial, they have to re‐consent by signing the informed consent document. For adult trial teams this might be an additional hurdle. However, as anecdotally shown in paediatric trials, well‐trained trial teams can successfully carry out the consent and assent processes.

Depending on country policy, some structural level adjustments would be beneficial to make inclusion of adolescents into clinical trials possible. For example in South Africa, a 12‐year‐old can consent to medical treatment without caregiver consent but cannot consent to participate in research. A stage 3 trial would benefit from including younger children for medical treatment, but other countries may not have this policy, which makes it difficult to manage the participants of the same age in other countries. Discussing this difficulty with key stakeholders could help determine specific country considerations and solutions.

Lastly, there are family and social‐cultural considerations for protecting adolescents from harm, especially in trials involving highly sensitive topics for families and their communities (sexual behaviour, gender identity, disclosure, etc.) [35]. Educating Institutional Review Boards and investigators about special regulatory protection can facilitate including adolescents in adult phase 3 clinical trials. Institutional Review Board committees play a critical role in advancing research for all populations and discussing these issues can overcome barriers to including adolescents in phase 3 adult clinical trials for adults.

### 2.8 Designing specific clinical trial materials for adolescents

In a discussion about conducting HIV preventive vaccine trials with adolescents Mc‐Clure *et al* [29] recommend designing protocols in close consultation with local community leaders and adolescent consultants. Using recruitment materials and clinic sites that are friendly, attractive and accessible to adolescents also can help improve participation.

Penta Foundation has supported the creation of Youth Trial Boards in Uganda, Zimbabwe, South Africa and UK to develop a youth‐centred approach for meaningful involvement of young patients in clinical trials and studies. ODYSSEY (PENTA 20) was the first trial that used a Youth Trial Board [30]. This model has been used in a few new trials (e.g. D3, BREATHER‐plus, LATA) in children, adolescents and young people led by Penta and/or the Clinical Trials Unit at the University College of London [33].

### 2.9 Contraception in adolescence

Contraception, especially for women, is usually an inclusion criterion particularly in clinical trials for new drugs or vaccines. Adolescents’ reluctance to disclose whether they are sexually active, is one of the many barriers which hinders their access to contraception [36]. Also, there is a lack of training among paediatricians and adult physicians on age‐appropriate sexual and reproductive health counselling [36].

In most settings, contraceptive use is governed by specific country policies. Therefore, enrolment of adolescent girls also depends on the country policy that dictates the age when adolescent girls can use contraceptives. Especially in resource‐limited settings key issues pertaining to the use of contraceptives in adolescent girls such as cultural norms, religious beliefs, community perspectives and stigma (for HIV and contraceptive use), might pose major enrolment concerns and could be addressed during the trial planning phase [31]. Raising community awareness and education is also crucial [32].

## 3 CONCLUSIONS

HIV‐positive adolescents remain at a disadvantage in terms of access to new drugs. Adolescents do not differ significantly from adults in weight, physiology, pharmacokinetics or adherence to treatment. Therefore, including ALHIV in initial phase 3 clinical trials with adults or in separate parallel studies could allow adolescents to benefit earlier from optimized ART. Coordinating research efforts with key stakeholders and sharing experiences on how to overcome perceived and real difficulties could help promote clinical trials among adolescents.

## COMPETING INTERESTS

The authors report no competing interests.

## AUTHORS’ CONTRIBUTIONS

The idea started at a WHO Paediatric Drug Optimization meeting (PADO 4) led by MP. PR and MP outlined the article. PR and DC wrote the first draft, which was reviewed by AT, MP and FV. The final draft was reviewed and approved by all authors.

## References

[1] Penazzato M , Gnanashanmugam D , Rojo P , Lallemant M , Lewis LL , Rochi F , et al. Optimizing research to speed up availability of pediatric antiretroviral drugs and formulations. Clin Infec Dis. 2017;64:1597–603. 29190337 10.1093/cid/cix194 PMC5927327

[2] FDA . Pediatric HIV Infection: drug product development for treatment, Guidance for industry. 2019 [cited 2020 Jan 8]. Available at: https://www.fda.gov/media/113319/download

[3] Weiss AR , Hayes‐Lattin B , Kutny MA , Stock W , Stegenga K , Freyer DR . Inclusion of adolescents and young adults in cancer clinical trials. Semin Oncol Nurs. 2015;31:197–205. 26210198 10.1016/j.soncn.2015.05.001 PMC5520996

[4] Sterling TR , Villarino ME , Borisov AS , Shang N , Gordin F , Bliven‐Sizemore EB , et al. Three months of rifapentine and isoniazid for latent tuberculosis infection. N Engl J Med. 2011;365:2155–66. 22150035 10.1056/NEJMoa1104875

[5] Venter WFD , Moorhouse M , Sohkela S , Fairlie L , Mashabane N , Masenya M , et al. Dolutegravir plus two different prodrugs of tenofovir to treat HIV. N Engl J Med. 2019;381:803–15. 31339677 10.1056/NEJMoa1902824

[6] Sawyer SM , Afifi RA , Bearinger LH , Blakemore SJ , Dick B , Ezeh AC , et al. Adolescence: a foundation for future health. Lancet. 2012;379:1630–40. 22538178 10.1016/S0140-6736(12)60072-5

[7] Kelley AE , Schochet T , Landry CF . Risk taking and novelty seeking in adolescence: introduction to part I. Ann N Y Acad Sci. 2004;1021:27–32. 15251871 10.1196/annals.1308.003

[8] UNAIDS . UNAIDS report, data sheet. [accessed 2020 Jun 8]; Available from: http://aidsinfo.unaids.org/2019

[9] UNAIDS . Start free stay free AIDS free 2019 report. Geneva: UNAIDS; 2019.

[10] Marshall WA , Tanner JM . Growth and physiological development during adolescence. Ann Rev Med. 1968;19:283–300. 4297619 10.1146/annurev.me.19.020168.001435

[11] Lu H , Rosenbaum S . Developmental pharmacokinetics in pediatric populations. J Pediatr Pharmacol Ther. 2014;19:262–76. 25762871 10.5863/1551-6776-19.4.262 PMC4341411

[12] Jacobson DL , Lindsey JC , Gordon CM , Moye J , Hardin DS , Mulligan K , et al. Total body and spinal bone mineral density across Tanner stage in perinatally HIV‐positive and uninfected children and youth in PACTG 1045. AIDS. 2010;24:687–96. 20168204 10.1097/QAD.0b013e328336095d PMC3154736

[13] Giacomet V , Mora S , Martelli L , Merlo M , Sciannamblo M , Vigano A . A 12‐month treatment with tenofovir does not impair bone mineral accrual in HIV‐positive children. J Acquir Immune Defic Syndr. 2005;40:448–50. 16280700 10.1097/01.qai.0000184860.62189.c8

[14] Schtscherbyna A , Pinheiro MF , Mendonça LM , et al. Factors associated with low bone mineral density in a Brazilian cohort of vertically HIV‐positive adolescents. Int J Infect Dis. 2012;16:e872–8. 10.1016/j.ijid.2012.07.019 23031418

[15] Panel on Antiretroviral Guidelines for Adults and Adolescents . Guidelines for the use of antiretroviral agents in adults and adolescents with HIV. Department of Health and Human Services [cited 2020 Jan 10]. Available from: http://aidsinfo.nih.gov/contentfiles/lvguidelines/AdultandAdolescentGL.pdf

[16] Panel on Antiretroviral Therapy and Medical Management of Children Living with HIV . Guidelines for the use of antiretroviral agents in pediatric HIV infection [cited 2020 Jan 12]. Available from: http://aidsinfo.nih.gov/contentfiles/lvguidelines/pediatricguidelines.pdf

[17] WHO . Consolidated guidelines on the use of antiretroviral drugs for treating and preventing HIV infection: recommendations for a public health approach. 2nd ed [cited 2020 Apr 20]. Available from: https://www.who.int/hiv/pub/arv/arv‐2016/en/

[18] Penta Writing Group . Penta HIV first and second line antiretroviral treatment guidelines. 2019 [cited 2020 Apr 20]. Available from: https://penta‐id.org/hiv/treatment‐guidelines/

[19] Bollen P . WB‐PK1 results in children 20‐<25kg. CROI 2019, poster #830.

[20] Bleyer A , Budd T , Montello M . Adolescents and young adults with cancer: the scope of the problem and criticality of clinical trials. Cancer. 2006;107:1645–55. 16906507 10.1002/cncr.22102

[21] Ferrari A , Montello M , Budd T , Bleyer A . The challenge of clinical trials for adolescents and young adults with cancer. Pediatr Blood Cancer. 2008;50:1101–4. 18360838 10.1002/pbc.21459

[22] Moore CL , Kekitinwa A , Kaudha E . and the ODYSSEY Trial Team . ODYSSEY: design, current status, and baseline characteristics. 10th International Workshop on HIV Pediatrics. Amsterdam, July 20–21st, 2018; poster Number 34.

[23] Hume M , Lewis LL , Nelson RM . Meeting the goal of concurrent adolescent and adult licensure of HIV prevention and treatment strategies. J Med Ethics. 2017;43(12):857–60. 28507222 10.1136/medethics-2016-103600 PMC5685924

[24] Mills EJ , Nachega JB , Buchan I , Orbinski J , Attaran A , Singh S , et al. Adherence to antiretroviral therapy in sub‐Saharan Africa and North America. JAMA. 2006;296:679–90. 16896111 10.1001/jama.296.6.679

[25] Kim SH , Gerver SM , Fidler S , Ward H . Adherence to antiretroviral therapy in adolescents living with HIV: systematic review and meta‐analysis. AIDS. 2014;28:1945–56. 24845154 10.1097/QAD.0000000000000316 PMC4162330

[26] BREATHER (PENTA 16) Trial Group . Weekends‐off efavirenz‐based antiretroviral therapy in HIV‐positive children, adolescents, and young adults (BREATHER): a randomised, open‐label, non‐inferiority, phase 2/3 trial. Lancet HIV. 2016;3:e421–30. 10.1016/S2352-3018(16)30054-6 PMC4995440 27562743

[27] Mystakidou K , Panagiotou I , Katsaragakis S , Tsilika E , Parpa E . Ethical and practical challenges in implementing informed consent in HIV/AIDS clinical trials in developing or resource‐limited countries. J Soc Aspects HIV/AIDS. 2009;6:46–57. 10.1080/17290376.2009.9724930 19936406

[28] Bwakura‐Dangarembizi M , Musesengwa R , Nathoo KJ , Takaidza P , Mhute T , Vhembo T . Ethical and legal constrains to children´s participation in research in Zimbabwe: experiences from the multicenter pediatric HIV ARROW trial. BMC Med Ethics. 2012;13:17. 22818109 10.1186/1472-6939-13-17 PMC3521203

[29] Mc‐Clure CA , Gray G , Rybczyk GK , Wright PF . Challenges to conducting HIV preventative vaccine trials with adolescents. J Acquir Immune Defic Syndr. 2004;36:726–33. 15167292 10.1097/00126334-200406010-00010

[30] Conway M . Nothing about us without us. 3rd International Workshop on HIV and adolescence. Nairobi, Kenya, Poster 40. 2019.

[31] Fairlie L , Waitt C , Lockman S , Moorhouse M , Abrams EJ , Clayden P , et al. Inclusion of pregnant women in antiretroviral drug research: what is needed to move forwards? J Int AIDS Soc. 2019;22:e25372. 31529598 10.1002/jia2.25372 PMC6747006

[32] Sullivan KA , Little MO , Rosenberg NE , Zimba C , Jaffe E , Gilbert S , et al. Women's views about contraception requirements for biomedical research participation. PLoS One. 2019;14:e0216332. 31067273 10.1371/journal.pone.0216332 PMC6505940

[33] Bernays S , Paparini S , Seeley J , Kihika SN , Gibb D , Rhodes T . Qualitative study of the BREATHER trial (Short Cycle antiretroviral therapy): is it acceptable to young people living with HIV. BMJ Open. 2017;7:e012934. 10.1136/bmjopen-2016-012934 PMC5318557 28213595

[34] Dybul M , Fauci AS , Bartlett JG , Kaplan JE , Pau AK . Guidelines for using antiretroviral agents among HIV‐infected adults and adolescents: the panel on clinical practices for treatment of HIV. Ann Int Med. 2002;137(5_Part_2):381–433. 12617573 10.7326/0003-4819-137-5_part_2-200209031-00001

[35] Field MJ , Behrman RE , Institute of Medicine (US) Committee on Clinical Research Involving Children . The Necessity and Challenges of Clinical Research Involving Children. In Ethical Conduct of Clinical Research Involving Children 2004. National Academies Press (US). 20669469

[36] Thongmixay S , Essink DR , Greeuw TD , Vongxay V , Sychareun V , Broerse JE . Perceived barriers in accessing sexual and reproductive health services for youth in Lao People’s Democratic Republic. PLoS One. 2019;14:e0218296. 31661486 10.1371/journal.pone.0218296 PMC6818758

